# Hepatitis C Virus Infection Upregulates Plasma Phosphosphingolipids and Endocannabinoids and Downregulates Lysophosphoinositols

**DOI:** 10.3390/ijms24021407

**Published:** 2023-01-11

**Authors:** Diren Beyoğlu, Stephanie Schwalm, Nasser Semmo, Andrea Huwiler, Jeffrey R. Idle

**Affiliations:** 1 Department of Pharmaceutical Sciences, College of Pharmacy and Health Sciences, Western New England University, Springfield, MA 01119, USA; 2 Hepatology Research Group, Department of BioMedical Research, University of Bern, CH-3008 Bern, Switzerland; 3 Pharmazentrum Frankfurt/ZAFES, Institute of General Pharmacology and Toxicology, University Hospital, Goethe University Frankfurt am Main, D-60590 Frankfurt am Main, Germany; 4 Institute of Pharmacology, Inselspital, INO-F, University of Bern, CH-3010 Bern, Switzerland

**Keywords:** hepatitis C virus, metabolomics, endocannabinoidome, fatty acid amide, GPR55, FAAH1, sphingosine 1-phosphate, SPHK1, lysophosphatidylinositol, bioactive lipid

## Abstract

A mass spectrometry-based lipidomic investigation of 30 patients with chronic hepatitis C virus (HCV) infection and 30 age- and sex-matched healthy blood donor controls was undertaken. The clustering and complete separation of these two groups was found by both unsupervised and supervised multivariate data analyses. Three patients who had spontaneously cleared the virus and three who were successfully treated with direct-acting antiviral drugs remained within the HCV-positive metabotype, suggesting that the metabolic effects of HCV may be longer-lived. We identified 21 metabolites that were upregulated in plasma and 34 that were downregulated (*p* < 1 × 10^−16^ to 0.0002). Eleven members of the endocannabinoidome were elevated, including anandamide and eight fatty acid amides (FAAs). These likely activated the cannabinoid receptor GPR55, which is a pivotal host factor for HCV replication. FAAH1, which catabolizes FAAs, reduced mRNA expression. Four phosphosphingolipids, d16:1, d18:1, d19:1 sphingosine 1-phosphate, and d18:0 sphinganine 1-phosphate, were increased, together with the mRNA expression for their synthetic enzyme SPHK1. Among the most profoundly downregulated plasma lipids were several lysophosphatidylinositols (LPIs) from 3- to 3000-fold. LPIs are required for the synthesis of phosphatidylinositol 4-phosphate (PI4P) pools that are required for HCV replication, and LPIs can also activate the GPR55 receptor. Our plasma lipidomic findings shed new light on the pathobiology of HCV infection and show that a subset of bioactive lipids that may contribute to liver pathology is altered by HCV infection.

## 1. Introduction

Hepatitis C virus (HCV) infection is a major cause of liver disease, including steatosis, fibrosis, and hepatocellular carcinoma. However, the mechanisms promoting liver disease progression by HCV are incompletely understood. In recent years, there has been focused research interest in the viral dependency upon various lipid metabolic processes in the multiple stages of the HCV life cycle [1]. A large international effort involving over 20 institutions has produced a proteogenomic atlas of HCV infection by the analysis of human hepatoma-derived Huh7.5.1 cells persistently infected with HCV [2]. From the identification of 21,950 mRNAs and 8297 proteins, a strongly impaired peroxisomal function was deduced, together with aberrations of fatty acid and bile acid metabolism [2]. In particular, the decreased β-oxidation from C_18_ to C_26_ in very long-chain fatty acids (VLCFAs) was reported. These observations of the impaired peroxisomal function of HCV-infected hepatocyte-like cells were confirmed in the livers of HCV-infected patients, in stark contrast to chronic hepatitis B virus (HBV)-infected patient livers, which display an increased peroxisomal function [2]. In addition, the cell membrane composition is altered in cells that replicate HCV RNA. For example, cholesterol and phosphatidylcholines accumulate in the endoplasmic reticulum during HCV infection [3], while fatty acid synthesis is attenuated, and β-oxidation increases in the liver cells [4]. Circulating HCV particles are physically associated with lipoproteins in infectious hybrid lipid aggregates known as lipoviral complexes, which bear a greater similarity to low-density lipoprotein (LDL) and very low-density lipoprotein (VLDL) particles than to a virion, with a composition of cholesterol, triglycerides, phosphatidylcholines, phosphatidylethanolamines, lysophosphatidylcholines, and sphingolipids [5,6]. These hijacked complexes appear to conceal viral epitopes to escape immune surveillance and utilize lipoprotein receptors for attachment and entry into hepatocytes [5]. Moreover, cytosolic lipid droplets formed during the HCV infection of hepatocytes were reported 40 years ago [7]. HCV replication requires numerous components of lipid metabolism and leads to the dysregulation of lipid homeostasis, which results in triglyceride accumulation in the liver [8]. The accumulated literature shows that HCV entry, replication, maturation, and egress depend upon various elements of lipid metabolism [1,5].

The diagnosis of the fatty liver occurs commonly in HCV-infected patients. It is believed that this may be due to the impaired secretion of VLDL, leading to reduced serum LDL [6]. Clearly, the metabolic pathways involving the aforementioned multiple classes of lipids that comprise lipoproteins may be affected in the liver. The two most abundant lipid classes in LDL comprise the sphingolipids sphingomyelin (SM) and ceramide (CER) [9]. Given the relationship between lipoproteins and HCV replication and cellular egress, it was not surprising that SM was involved in these processes. It has recently been reported for the first time that SM was essential for the biosynthesis of double-membrane vesicles found in the replication factories that are the sites of HCV RNA production [10].

This knowledge of the relationship between HCV infection and lipid metabolism derives mainly from the study of cell cultures and investigations targeted at specific lipid classes. Using a local biobank that contains frozen patient biofluids and liver biopsies, we decided to conduct an untargeted mass spectrometry-based metabolomic investigation of HCV infection. We report here multiple lipid alterations in HCV-positive patient plasma compared to the HCV-negative controls, including the upregulation of multiple fatty acid amides and phosphosphingolipids and the downregulation of a raft of phospholipids, of two galactoceramides, together with the quenching of ceramide 1-phosphate. These HCV-induced metabolic aberrations cast further light on the hepatic metabolic processes set in train by HCV.

## 2. Results

### 2.1. Ultraperformance Liquid Chromatography-Electrospray Ionization-Quadrupole Time-of-Flight Mass Spectrometry (UPLC-ESI-QTOFMS)-Based Lipidomics

**Figure 1:**
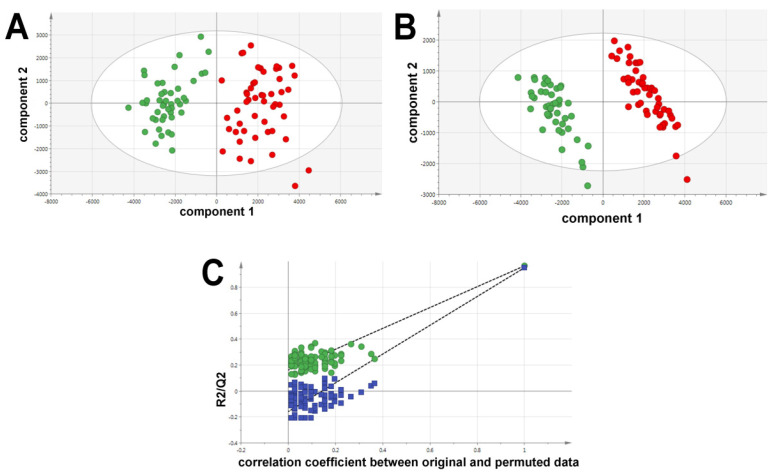
Multivariate data analysis of HCV-positive patient plasmas (red) and HCV-negative control plasmas (green). Samples were analyzed in duplicate. (**A**) PCA scores plot showing complete separation of the HCV-positive and negative plasmas. (**B**) PLS-DA scores plot showing enhanced resolution of the HCV-positive and negative plasmas. (**C**) Leave-one-out cross validation of the PLS-DA model with 200 permutations for R2 (green) and Q2 (blue).

The multivariate data analysis of the ESI+ data derived from the comparison between HCV-positive patients and control plasma by UPLC-ESI-QTOFMS is displayed in Figure 1. The unsupervised principal components analysis (PCA; Figure 1A) shows a complete separation between HCV-positive and HCV-negative plasma samples, indicating discrete metabolic phenotypes for each sample group. A supervised partial least squares-discriminant analysis (PLS-DA; Figure 1B) of the two sample groups further improved their resolution. To determine if the data were over-modeled by PLS-DA, leave-one-out cross-validation was performed (Figure 1C). R2 (goodness of fit) and Q2 (goodness of prediction) were coincident with values close to the maximum of 1.0, indicating the high predictive accuracy of the model. After 200 permutations, R2 declined to <0.2 and Q2 to <0, showing that the data were not overfitted to the PLS-DA model [11]. It is interesting to note that three patients for whom the viral infection resolved spontaneously and three patients who were virus-free after chemotherapy with direct-acting antivirals all remained within the HCV-positive metabotype [12] and did not migrate to join the HCV-negative control metabotype. This indicates that the effects of HCV infection on lipid metabolism may be long-lived.

In order to interrogate the metabolic basis for the appearance of two distinct metabotypes for HCV-positive and HCV-negative plasma samples, UPLC-ESI-QTOFMS data were analyzed with Progenesis QI software. The database mining of *m*/*z* values, isotope abundances, and fragmentation patterns for each positive and negative ion (18,599 generated), together with a comparison of authentic lipid standards (see below), yielded the findings shown in Table 1. Twenty-one identified metabolites were elevated, and 34 were diminished in HCV-positive plasma relative to the HCV-negative control plasma.

**Figure 2:**
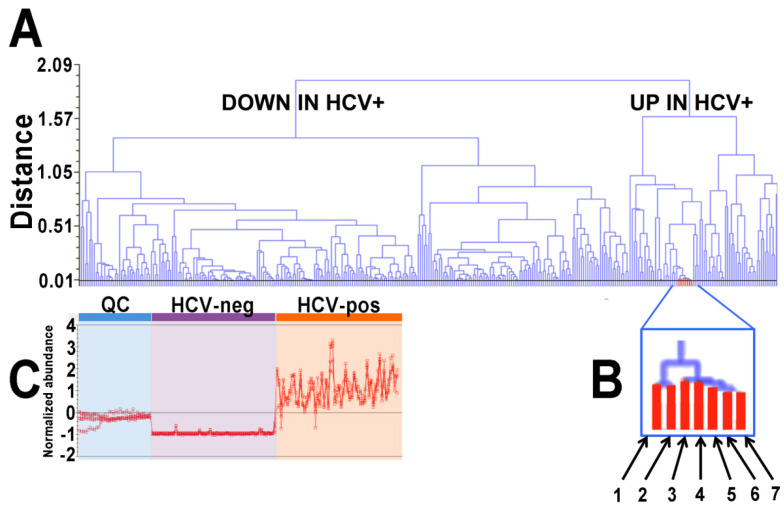
(**A**) Up- and down-regulated plasma metabolites in HCV-positive patients displayed in a dendrogram. (**B**) A single node contained all seven fatty acid amides detected by UPLC-ESI-QTOFMS, as follows: 1, decanamide; 2, dodecanamide; 3, palmitamide; 4, stearamide; 5, palmitoleamide; 6, linoleamide; 7, eicosatetraenamide. (**C**) Abundance profiles for the seven FAAs across 21 QC samples, 30 HCV-negative samples, and 30 HCV-positive samples.

These 55 identified metabolites were statistically significant (*p* < 1 × 10^−16^ to 0.0002) and altered (≥2-fold) in HCV-positive patient plasma. Six were derived from negative ions (M − H^−^ and M + Cl^−^) and 49 from positive ions (M + H^+^, M + Na^+^ and M + H − H_2_O^+^). A clear pattern in the upregulated metabolites emerged, with seven fatty acid amides (FAAs) and four phosphosphingolipids (PSLs), all statistically significantly increased in the HCV-positive plasma (Table 1). The up- and down-regulated metabolites, including the seven related FAAs, can be seen in a dendrogram (Figure 2). Note that considerably more metabolites were downregulated than upregulated.

**Figure 3:**
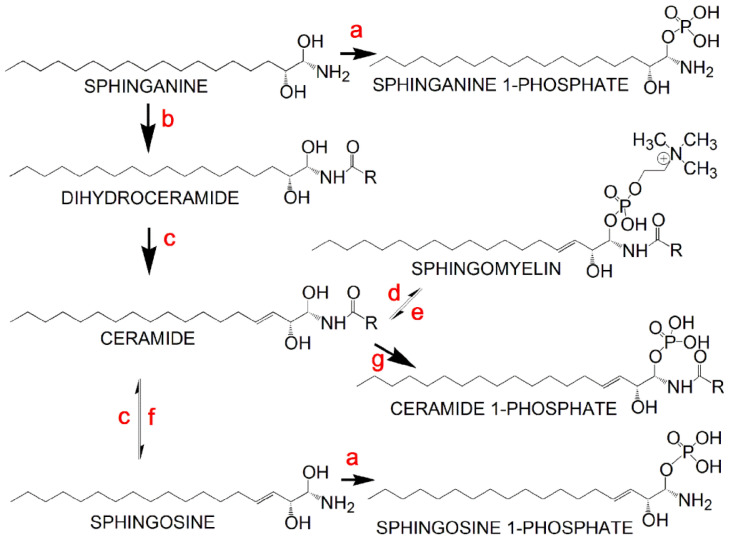
Metabolic interconversions of the principal sphingolipids: a, sphingosine kinase (EC 2.7.1.91); b, ceramide synthase (EC 2.3.1.299); c, dihydroceramide desaturase (EC 1.14.19.17); d, sphingomyelin synthase (EC 2.7.8.27); e, sphingomyelin phosphodiesterase (EC 3.1.4.12); f, ceramidase (EC 3.5.1.23); g, ceramide kinase (EC 2.7.1.138).

In addition to the FAAs, two fatty acid ethanolamides, anandamide, derived from arachidonic acid, and LTB_4_-ethanolamide, together with *N*-linoleoyltaurine, were also upregulated in HCV-positive plasmas. LTB_4_-ethanolamide is a dual antagonist of BLT and TRPV1 receptors with anti-inflammatory activity [13]. Anandamide (*N*-arachidonylethanolamine) was the first endocannabinoid to be discovered [14] and binds both CB_1_ and CB_2_ cannabinoid receptors [15]. *N*-Linoleoyltaurine was the most abundant member of the *N*-acyltaurines reported in mouse plasma: a group of lipid messengers that improve postprandial glucose regulation [16]. Four phosphosphingolipids were statistically significantly elevated (2.0- to 5.3-fold) in HCV-positive plasma, including three sphingosine 1-phosphates, d18:1, d16:1, and d19:1, together with sphinganine 1-phosphate d18:0. Sphingosine 1-phosphate (S1P) can act as a G protein-coupled receptor ligand and as a signaling molecule. S1P also plays a well-known part in cell signaling: the cell death/survival decision, immunomodulation, vascular integrity, and the pro-inflammatory response [17]. S1P mimics some of the profibrotic functions of TGF-β by the activation of Smad signaling, whereas sphinganine 1-phosphate (dhS1P) displays an opposing effect on TGF-β signaling [18]. The metabolic interconversion of the principal sphingolipids is represented in Figure 3.

Among the miscellaneous upregulated metabolites in HCV-positive plasmas was the dietary C9 dicarboxylic acid azelaic acid, which is an inhibitor of tyrosinase and various oxidoreductases [19]. In addition, the brain neurosteroid pregnenolone sulfate [20] and the *N*-glycosylation precursor dolichol phosphate [21] were both elevated. Finally, three phospholipid metabolites were enhanced, of which one, the infrequent phosphatidylserine PS(22:0/22:0) [22], was the highest elevated metabolite in the HCV-positive plasma (446-fold; Table 1).

**Figure 4:**
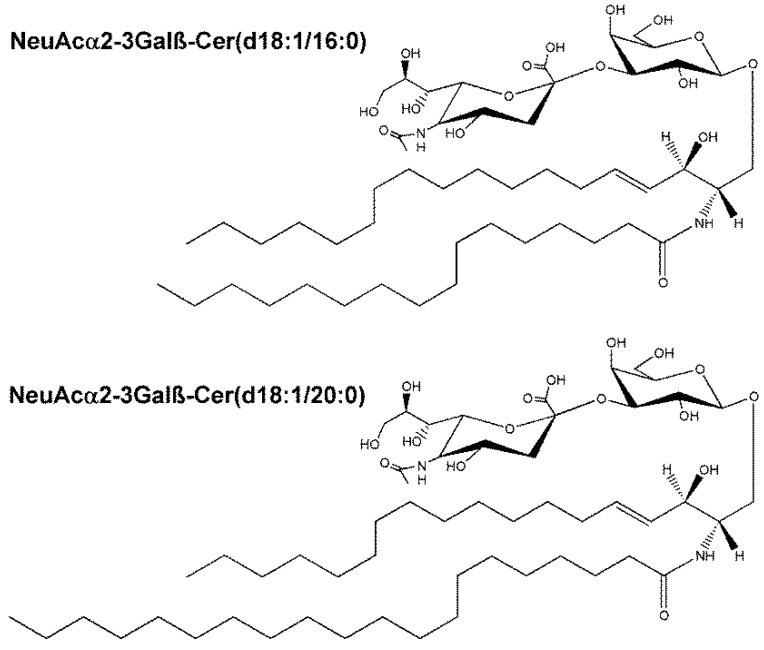
Two galactoceramides diminished in plasma of HCV-positive patients.

The downregulated metabolites in HCV-positive plasma included two triglycerides and 25 phospholipids (Table 1), of which two, lysophosphatidylserine LPS(17:1(9*Z*)) and lysophosphatidylinositol LPI(22:6), showed the greatest decline at 1011-fold and 2879-fold, respectively. In HCV-positive patient plasma the signal for the prosurvival molecule [23] *N*-acetylsphingosine 1-phosphate (C2 ceramide 1-phosphate) was extinguished. The antiproliferative metabolite [24] anandamide *O*-phosphate was diminished 2.4-fold in the HCV-positive plasma. Two primary bile acid conjugates, glycochenodeoxycholic acid 7-sulfate [25] and cholic acid glucuronide [26] were both significantly diminished by HCV. The leukotriene metabolite lipoxin C4 [27] and the two galactoceramides NeuAcα2-3Galβ-Cer(d18:1/16:0) and NeuAcα2-3Galβ-Cer(d18:1/20:0) (Figure 4) were diminished by HCV infection.

### 2.2. Gas Chromatography-Mass Spectrometry-Based Metabolomics

**Figure 5:**
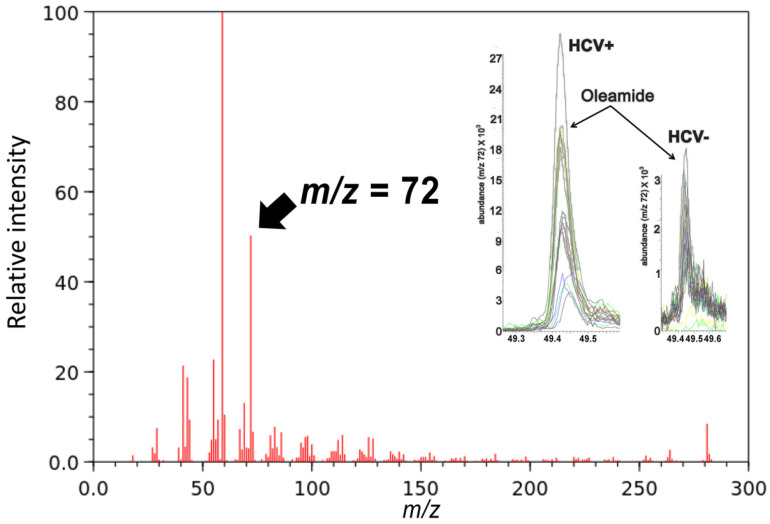
GC-MS spectrum of oleamide (FAA(18:1)). Insert shows superimposed single-ion peaks (*m*/*z* = 72) for 30 HCV-positive plasmas and 30 HCV-negative plasmas.

We have previously reported the results of a metabolomics investigation of HCV infection using the plasma and urine from HCV-positive patients and HCV-negative age- and sex-matched controls [28]. We reexamined the plasma data for evidence of FAAs and found that oleamide was six-fold elevated in HCV-positive plasma by monitoring the ion at *m*/*z* = 72 (Figure 5).

The total number of plasma FAAs found to be elevated by HCV was eight, 10:0, 12:0, 16:0, 16:1, 18:0, 18:1, 18:2, and 20:4 (Figure 2 and Figure 5).

### 2.3. Gene Expression Analysis by Quantitative RT-PCR (qRT-PCR)

In order to investigate the mechanism by which phosphosphingolipids and fatty acid amides were elevated in the plasma of HCV-positive patients, the expression of sphingosine kinase 1 (*SPHK1*) and 2 (*SPHK2*), S1P receptor 1 (*S1PR1*), 2 (*S1PR2*), and 3 (*S1PR3*), sphingomyelin phosphodiesterase 1 (*SMPD1*) and 2 (*SMPD2*), sphingosine 1-phosphate lyase 1 (*SGPL1*), ceramide kinase (*CERK*), peptidylglycine alpha-amidating monooxygenase (*PAM*), fatty acid amide hydrolase 1 (*FAAH1*) and 2 (*FAAH2*), acyl-CoA synthetase long-chain family member 3 (*ACSL3*), 4 (*ACSL4*) and 5 (*ACSL5*) were all determined by qRT-PCR in liver biopsies from HCV-positive patients and from HCV-negative autoimmune hepatitis patients.

**Figure 6:**
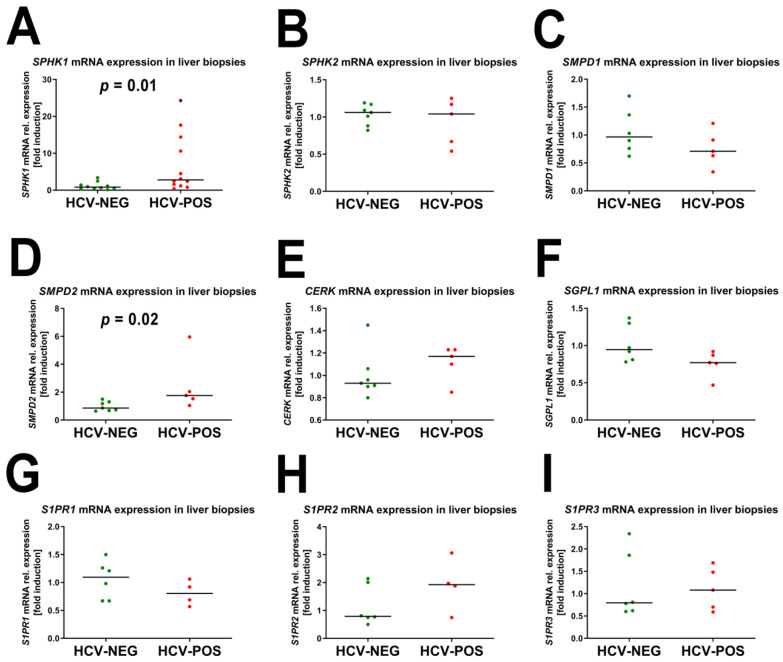
mRNA expression of genes involved in sphingolipid metabolism. (**A**) Sphingosine kinase 1 (*SPHK1*);(**B**) Sphingosine kinase 2 (*SPHK2*); (**C**) Sphingomyelin phosphodiesterase 1 (*SMPD1*); (**D**) Sphingomyelin phosphodiesterase 2 (*SMPD2*); (**E**) Ceramide kinase (*CERK*); (**F**) Sphingosine 1-phosphate lyase (*SGPL1*); (**G**) Sphingosine 1-phosphate receptor 1 (*S1PR1*); (**H**) Sphingosine 1-phosphate receptor 2 (*S1PR2*); (**I**) Sphingosine 1-phosphate receptor 3 (*S1PR3*).

The qRT-PCR findings for sphingolipids are shown in Figure 6.

There was a statistically significant 3.4-fold increase in *SPHK1* mRNA expression between HCV-positive and -negative liver biopsies (Figure 6), which correlated well with the mean 3.1-fold (2- to 5.3-fold) increase in HCV-positive patient plasmas compared to the HCV-negative control plasma (Table 1) for sphingosine phosphates and sphinganine phosphate, all formed by sphingosine kinase (Figure 3). Furthermore, a statistically significant median 2.5-fold increase in *SMPD2* mRNA expression was observed. Sphingomyelin phosphodiesterase (SMPD 1 and 2) produces ceramide, which is a precursor of sphingomyelin in the back reaction involving sphingomyelin synthase (mRNA expression not determined) (Figure 3). No other mRNA expression differences in Figure 6 showed statistical significance.

**Figure 7:**
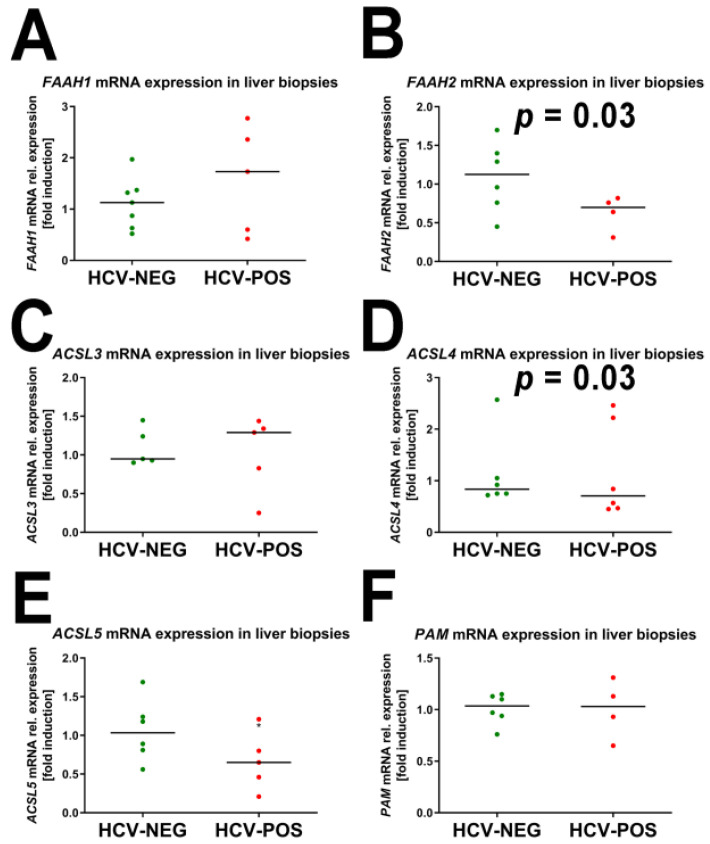
mRNA expression of genes involved in FAA metabolism. (**A**) Fatty acid amide hydrolase 1 (*FAAH1*); (**B**) Fatty acid amide hydrolase 2 (*FAAH2*); (**C**) Acyl-CoA synthetase long chain family member 3 (*ACSL3*); (**D**) Acyl-CoA synthetase long chain family member 4 (*ACSL4*); (**E**) Acyl-CoA synthetase long chain family member 5 (*ACSL5*); (**F**) Peptidylglycine alpha-amidating monooxygenase (*PAM*).

The qRT-PCR findings for FAAs are shown in Figure 7.

The mRNA expression of two genes related to FAA metabolism showed statistically significant differences (*p* = 0.03) between HCV-positive and HCV-negative liver biopsies. First, *FAAH2* expression showed a 1.7-fold decrease in HCV-positive liver biopsies (Figure 7). This finding is consistent with the elevated plasma concentrations of FAAs found in HCV-positive patients (Table 1). Moreover, *ACSL4*, which might be involved in the synthesis of FAAs, displayed a 1.8-fold decrease in the expression of HCV-positive biopsies. No other mRNA expression differences in Figure 7 showed statistical significance.

### 2.4. Correlation of mRNA Fold Induction with Viral Load

For all 15 gene expression analyses shown in Figure 6 and Figure 7, the correlation with viral load for each HCV-positive patient was investigated by Spearman rank correlation. The mRNA expression of only one gene (*CERK*) correlated statistically significantly with viral load (*r_s_* = 0.97; *p* = 0.03). Ceramide kinase produces ceramide 1-phosphate (C1P), which stimulates DNA synthesis and cell division and promotes cell survival [29,30].

## 3. Discussion

Our mass spectrometry-based lipidomic investigation of the metabolic consequences of HCV infection revealed in a PCA scores plot the existence of two distinct metabolic phenotypes in the plasma that distinguished 30 HCV-positive patients from age- and sex-matched HCV-negative blood donor controls. The PLS-DA model had high predictive accuracy, with R2 and Q2 values ≈of 1.0. Six of the patients in the HCV-positive group had cleared the virus from their plasma either spontaneously (n = 3) or after DAA therapy (n = 3) but did not revert to the HCV-negative metabotype. There are relatively few published data regarding the metabolic effects in HCV-positive patients who have undergone DAA therapy. One such study used NMR spectroscopy to estimate the comparative serum levels of ten small non-lipid metabolites, including four amino acids, in 67 HCV-positive patients before and after DAA therapy (12 weeks and 24 weeks sustained virological response, SVR12 and SVR24). Many of the metabolic differences were small and of marginal statistical significance. Focusing on 2-oxoglutarate (a TCA cycle intermediate) and 3-hydroxybutyrate (a product of valine catabolism), the authors claimed to have “revealed some alteration in metabolites” related to SVR responses [31]. A recent report of triglyceride profiles in sera from 177 HCV patients found that serum triglycerides were lower in male patients with cirrhosis than in male patients without cirrhosis after DAA therapy. Overall, triglyceride levels did not change in either sex after DAA therapy [32]: a finding consistent with our observations reported here. This same group investigated eight serum ceramide concentrations in 178 patients with chronic HCV infection and noted that most ceramides are carried on LDL, which rises after effective DAA therapy. Again, they reported differences in cirrhotic versus noncirrhotic patients, especially in the ratio of long-chain ceramides (such as d18:1; O2/18:0) to very long-chain ceramides (such as d18:1; O2/24:0) [6]. In our untargeted lipidomic analysis here, we did not observe changes in these specific ceramide molecules but did observe 2.8- to 3.4-fold diminished plasma concentrations for two galactoceramides, one containing a long-chain ceramide (d18:1/16:0) and the other a very long-chain ceramide (d18:1/20:0). However, the cirrhosis status of our biobanked plasmas was unknown.

The plasma concentrations of multiple classes of lipids were altered statistically significantly in HCV-positive patients versus HCV-negative controls. Most strikingly, we report here that eight FAAs were elevated highly statistically significantly (*p* < 1 × 10^−16^ to 0.0002) from 2- to 80-fold. Additionally, two *N*-acyl ethanolamides, anandamide (*N*-arachidonylethanolamine) and LTB_4_-ethanolamide, together with *N*-linoleoyltaurine, were all elevated in plasma by HCV infection. Interestingly, LPG(22:4(5*Z*,8*Z*,11*Z*,14*Z*)), better known as 2-arachidonylglycerol, a canonical endocannabinoid, was diminished in plasma ~50-fold by HCV infection. The upregulated 11 molecules belong to what is now referred to as the “endocannabinoidome”, which has expanded the original group of just two endocannabinoids, anandamide, and 2-arachidonylglycerol, to now contain various FAAs, *N*-fatty acyl-ethanolamines, -glycines, -taurines, -serines, -dopamines, and -serotonins [33,34]. These new endocannabinoids do not act on the two cannabinoid receptors CB_1_ and CB_2_ but rather on a range of other molecular targets, including G protein-coupled receptors (e.g., the novel cannabinoid receptor GPR55 [35]), nuclear receptors, and several enzymes and transporter proteins [34]. Of the 11 endocannabinoids that were elevated by HCV, there are sparse examples in the literature concerning many of them. For example, decanamide is only referred to as a minor metabolite of the bacterium *Pseudomonas* X2 when grown with n-decane as its only carbon source [36]. In contrast, dodecanamide, palmitamide, stearamide, oleamide, and linoleamide were all produced by HepG2 cells treated with extracts of the brown algae *Fucus vesiculosus* [37]. Dodecanamide has been reported as an intracellular metabolite in MCF-7 cells after doxorubicin-induced cell death [38] and with diminished levels in the serum of acute myeloid leukemia patients who responded well to chemotherapy with cytarabine plus anthracycline [39]. There exist more extensive studies in the literature on palmitamide, palmitoleamide, stearamide, oleamide, and linoleamide [37,40,41,42,43,44], but none in relation to HCV and the liver, with the exception of oleamide in our earlier metabolomics report on HCV-infected patients [28]. Therefore, our description of these seven FAAs elevated due to HCV infection is novel, which is additionally supported by our observation that the FAA catabolic enzyme FAAH2 showed a 40% decrease (*p* = 0.03) in mRNA expression due to HCV. Together with these upregulated FAAs, the canonical endocannabinoid anandamide was increased 4.3-fold. There are only a few reports on anandamide and HCV. Serum anandamide was elevated ~2-fold in chronic hepatitis B but not chronic hepatitis C [45]. In the cell culture, HCV replication increased cellular 2-arachidonylglycerol ~2-fold, but not anandamide [46]. Finally, a report from one of us (NS) showed that both anandamide and 2-arachidonylglycerol were elevated in the plasma but not in the liver tissues of HCV-positive patients. Additionally, hepatic FAAH mRNA expression was reduced in liver tissues [47], as we have reported here, but our data on 2-arachidonylglycerol contradict these earlier findings. Overall, we have reported here the upregulation in the plasma of HCV-infected patients: 11 members of the endocannabinoidome. The role of these lipids in HCV replication and egress is yet to be investigated.

Inoue and colleagues described a number of Huh-7 derived subclones in relation to both HCV replication efficiency and the expression of UDP-galactose:ceramide galactosyltransferase (UGT8; EC 2.4.1.62): a key enzyme in the synthesis of galactoceramides. The HuhTe6 subclone displayed a 4.9-fold decreased expression of *UGT8* than HuhTe4 cells but maintained a 100-fold greater HCV replication efficiency than HuhTe4 cells [48], pointing to an inverse relationship between HCV replication and *UGT8* mRNA expression. This finding is consistent with the observation reported here that HCV-positive patients have between 2.8- and 3.4-fold decreased plasma concentrations of NeuAcα2-3Galβ-Cer(d18:1/16:0) and NeuAcα2-3Galβ-Cer(d18:1/20:0) than HCV-negative controls.

Among the largest lipidomic changes due to HCV infection, this involved a number of lysophosphoinositol (LPI) molecules, which were diminished in the plasma between ~3- and ~3000-fold and, in one case [LPI(20:2(11*Z*,14*Z*))], was completely extinguished in the plasma. A second ω-6 LPI [LPI(22:4(7*Z*,10*Z*,13*Z*,16*Z*))] was reduced >150-fold, with two ω-3 LPIs [LPI(20:5(5*Z*,8*Z*,11*Z*,14*Z*,17*Z*)) and LPI(22:6(4*Z*,7*Z*,10*Z*,13*Z*,16*Z*,19*Z*))] which decreased to >450- and ~3000-fold, respectively. A single saturated LPI(21:0) was reduced ~3-fold. Phosphoinositides (PIPs) are integral components of certain intracellular membranes and act as docking sites for protein recruitment. HCV uses viral replication complexes and promotes the concentration of augmented pools of phosphatidylinositol 4-phosphate (PI4P) near the sites at the endoplasmic reticulum. It does this by activating phosphatidylinositol 4-kinase alpha (PI4Kα; EC 2.7.1.67). The inhibition of this enzyme abolishes HCV replication [49]. The generation of these PI4P pools necessarily depletes cellular phosphatidylinositols, which corroborates our finding of diminished phosphatidylinositols in the plasma. Phagocytic cells, such as monocytes and macrophages, incorporate large amounts of fatty acids into PIPs: in particular, polyunsaturated fatty acids (PUFAs) such as arachidonic acid. The synthesis of 1,2-diarachidonoyl-glycero-3-phosphoinositol was a major phosphoinositol formed in the monocytes [50]. It is likely that hepatic Kupffer cells, the body’s most abundant tissue-resident macrophage, would also generate PIPs comprising PUFAs. It would appear that HCV forces the liver to induce PI4Kα, incorporating PUFA-containing PIPs into the double-membrane vesicles of viral replication factories [10]. In accordance with our plasma lipidomic findings, it is apparent that the PUFA-containing PIPs used may comprise the ω-3 fatty acids EPA (20:5) and DHA (22:6). It is expected that these VLCFAs will be more bioavailable due to the peroxisomal β-oxidation malfunction in hepatocytes caused by HCV [2].

Furthermore, LPIs have been found to activate the orphan receptor GPR55 [51,52] that also binds the endocannabinoids anandamide, 2-arachidonylglycerol, *N*-palmitoylethanolamide, and *N*-oleoylethanolamide [35]. LPI signaling cascades through GPR55 and is involved in cell proliferation, migration, survival, and tumorigenesis [53]. It has been reported that GPR55 is a pivotal host factor for HCV replication by downregulating the expression of interferon-stimulated genes, such as *ISG15*, *Mx1*, and *IFITM1*, that comprise part of the cellular antiviral response [54]. It is possible, therefore, to envisage a mechanistic connection between HCV infection and the elevated plasma levels of the endocannabinoids, including FAAs.

Three sphingosine 1-phosphates (S1Ps), d16:1, d18:1, and d19:1, were increased in HCV-positive plasma from two- to five-fold. In addition, d18:0 sphinganine 1-phosphate was similarly increased. Of the nine genes related to sphingolipid metabolism that we assayed in liver tissues by qRT-PCR, only two were altered by HCV infection, *SPHK1* (3.4-fold increased) and *SMPD2* (2-fold increased). Bovine viral diarrhea virus (BVDV), a close relative of HCV, inhibited the catalytic activity of SPHK1, which was seen as beneficial for DVCV replication, and it was further proposed that this virus specifically targeted SPHK1 to regulate its catalytic activity [55]. HCV chronic infection was shown to induce specific genome-wide changes in mRNA and protein expression, which persisted after the treatment of patients achieved SVR with either DAA therapy or interferons. One such change related to increased SPHK1 expression with HCC risk [56]. The persistent post-therapy gene expression changes correlate with our finding that patients who had spontaneously eliminated HCV or had undergone DAA chemotherapy retained the metabolomic phenotype of actively infected patients. Finally, the polarization of M2 macrophages into M2a, M2b, and M2c subtypes has been investigated with respect to HCV. M2c macrophages polarized from patients with chronic hepatitis C, expressed lower levels of SPHK1 than those from healthy controls [57]. Our plasma lipidomic findings of elevated d16:1, d18:1, and d19:1 S1P concentrations in HCV patients reflect holistic SPHK1 and SPKH2 activities, not simply that which is in M2c macrophages. SPHK1 is expressed in multiple human tissues (https://www.proteinatlas.org/ENSG00000176170-SPHK1/tissue, accessed on 27 October 2022). SPHK1 is known to be upregulated by classical pro-inflammatory stimuli that also drive M1 polarization. However, its detailed regulatory role in macrophage polarization is not yet clear. In contrast, the inhibition or deletion of Sphk2 was shown to promote M2 macrophage polarization [58]. So far, it is not possible to discriminate between Sphk1- and Sphk2-produced S1P in cells or tissues. Based on our data that SPHK1 mRNA and S1P are coordinately enhanced in HCV patients, it is tempting to speculate that SPHK1 is the key enzyme for S1P generation. However, it cannot be excluded that SPHK2 plays a role too since both of the enzymes are known to also be regulated post-transcriptionally. It is worth noting that d19:1 S1P is a rare sphingosine but would have the same *m*/*z* value as the isomers iso-d19:1 S1P and anteiso-d19:1 S1P, which are probably less common. However, iso- and anteiso-sphingosines have been described in mammalian tissues [59,60].

Another important aspect of HCV infection is the occurrence of T cell exhaustion which reduces the protective immunity during chronic infection. Exhausted T cells are defined by the expression of multiple co-inhibitory molecules among those of PD-1, Lag3, CD244 (2B4), and CD39 [61,62]. Interestingly, it was recently shown that S1P could contribute to exhaustion in CD8+ cytotoxic T cells by upregulating PD-L1 expression [63]. Moreover, CD8+ T cell expansion was abolished by the S1P4 receptor blockade [64]. This potential novel regulatory role of the Sphk1/S1P axis on T cell exhaustion clearly requires further validation. One limitation of our study was the lack of patient details for the Biobank in both plasma and liver tissue samples. However, we have used cutting-edge UPLC-ESI-QTOFMS procedures to analyze both 30 patients and 30 control plasmas. Coupled with this were advanced chromatogram and spectra analysis, contemporary multivariate data analyses, and gene expression analyses. This has allowed us to report here a number of novel findings regarding chronic HCV infection and cast new light on the underlying mechanisms of HCV infection.

## 4. Materials and Methods

### 4.1. Authentic Standards

The following lipids were obtained from Sigma-Aldrich (Buchs, Switzerland): oleamide, stearamide, glyceryl tristearate, glyceryl trimyristate, glyceryl tripalmitate, palmitoleic acid, oleic acid, and cholesterol. The following lipids were obtained from Avanti Polar Lipids (Alabaster, AL, USA): PC(16:0/20:4), PC(17:0/17:0), PC(18:0/18:0), PC(18:2/18:2), PC(20:4/20:4), PC(22:6/22:6), PC(19:0/19:0), PC(16:0/16:0), LPC(16:0), LPC(17:0), LPC(18:1), LPC(18:0), LPA(17:0), LPA(18:0), LPA(20:4), LPA(18:1), LPA(14:0) and LPA(16:0). Standards were dissolved in propan-2-ol in groups of 10 to 20 in the concentration range 10 to 80 μM for analysis by UPLC-ESI-QTOFMS (see below). Oleamide was analyzed by GC-MS (see below).

### 4.2. Subjects and Clinical Materials

Thirty patients who were positive for HCV were identified in the Bern Hepatology Biobank. All participants gave their written informed consent to donate blood to the biobank, and the study was conducted according to the World Medical Association Declaration of Helsinki. Patients had a mean (±SD) age of 49.2 ± 11.1 years and comprised 15 males (M) and 15 females (F). Twenty-four subjects had an active viral infection (median viral load 478,059 U/mL), three had spontaneously resolved, and three were HCV eliminated after antiviral therapy with an undetectable virus. Control EDTA plasmas were obtained from the local blood bank from voluntary blood donors from all over Switzerland (15 M, 15 F, aged 41 to 55 years). All samples tested negative for the presence of antibodies and/or antigens for HIV, HCV, and HBV. All samples were also negative for syphilis. HIV, HBV, and HCV. PCR testing was also negative for all the control samples. The HCV-positive patients and HCV-negative controls did not differ statistically significantly by age or gender.

Percutaneous needle liver biopsy material from additional HCV-positive patients (n = 6) and HCV-negative patients with autoimmune hepatitis (n = 6) was also obtained from the Bern Hepatology Biobank.

### 4.3. RNA Extraction and Quantitative PCR Analysis

Liver biopsies frozen in liquid nitrogen were homogenized in a Mikro-Dismembrator S (Sartorius Stedim Biotech GmbH, Dietikon, Switzerland) at 3000 rpm for 30 s and resuspended in 1.5 mL of TRIZOL™ reagent (Sigma-Aldrich, Steinheim, Germany). The total RNA was extracted according to the manufacturer’s protocol and used for reverse transcriptase polymerase chain reaction (RT-PCR; RevertAid™ first strand cDNA synthesis kit Thermo Fisher Scientific, Waltham, MA, USA) utilizing a random hexamer primer for amplification. A TaqMan quantitative PCR assay (Applied Biosystems, Thermo Fisher Scientific, Waltham, MA, USA) was performed using the Applied Biosystems 7500 Fast Real-Time PCR System. TaqMan^®^ gene expression assays and qPCR Low Rox Mix were from Life Technologies (Darmstadt, Germany). The following TaqMan^®^ gene expression assays were used: hSPHK1: Hs01116530_g1; hSPHK2:Hs00219999_m1; hS1PR1:Hs00173499_m1; hS1PR2: Hs00244677_s1; hS1PR3:Hs01015603_s1; hSMPD1:Hs03679347_g1; hSPNS2: Hs1390449_g1; hCERK:Hs00368483_m1; hSGPL1:Hs00187407_m1; hPAM: Hs00168596_m1; hFAAH1:Hs01038664_m1; hFAAH2:Hs00398732_m1; hACSL3: Hs00244853_m1; hACSL4:Hs00244871_m1; hACSL5:Hs00212106_m1 (Life Technologies, Darmstadt, Germany).

The cycling conditions were: 95 °C for 15 min (1 cycle), 95 °C for 15 s, and 60 °C for 1 min (40 cycles). The threshold cycle (C_t_) was calculated by the instrument’s software (7500 Fast System SDS Software version 1.4). The analysis of the relative mRNA expression was performed using the ΔΔC_t_ method. Eukaryotic 18S ribosomal RNA: Hs99999901_s1 (Life Technologies, Darmstadt, Germany) was used for normalization.

### 4.4. Ultraperformance Liquid Chromatography-Electrospray Ionization-Quadrupole Time-of-Flight Mass Spectrometry (UPLC-ESI-QTOFMS)

For lipidomic analysis, plasma (50 μL) was added to cold propan-2-ol (500 μL) containing 5 μM of LPC(17:0) as an internal standard. The solution was vortexed for 30 s, then centrifuged at 18,000× *g* for 5 min to remove precipitated proteins, and the clear supernatants were transferred to UPLC injection vials. UPLC-ESI-QTOFMS analysis was conducted in the Clinical Metabolomics Center, Inselspital, Bern, using a Waters Acquity CSH 1.7 μm C18 column (2.1 × 100 mm) under the following conditions: solvent A, acetonitrile/water (60:40 *v*/*v*) containing 10 mM ammonium acetate and 0.1% formic acid; solvent B, propan-2-ol/acetonitrile (90:10 *v*/*v*) containing 10 mM ammonium acetate and 0.1% formic acid. The gradient was 60% A to 57% A at 2 min, to 50% A at 2.1 min (ballistic gradient), to 46% A at 12 min, to 30% A at 12.1 min (ballistic gradient), to 1% A at 18 min before returning to initial conditions at 18.5 min, and equilibration for 2 min. The flow rate was 0.4 mL/min, and the column was maintained at 55 °C. Mass spectrometry on UPLC eluates was conducted in positive (ESI+) and negative (ESI-) modes using a Waters Synapt HDMS QTOFMS, scanning 50–1000 amu at a rate of 3.3 scans/s. The capillary voltage was 3 kV, the source temperature was 120 °C, the sampling cone was 30 V, and the nitrogen desolvation gas flow was 850 L/h at 400 °C. The total run time was 18 min. Samples were randomized and included 21 pooled QC samples, blanks, and a standard mixture to monitor instrument stability.

Data were collected in a continuum mode for retention time (RT) from 0.5 to 17 min, and the raw chromatographic data were imported into Progenesis QI 2.1 software (Waters Corporation, Wilmslow, UK) for visualization of chromatograms as ion intensity maps, chromatogram alignment, peak picking, deconvolution, and normalization. In the ESI+ mode, the following adducts were considered when solving empirical formulae from accurate mass determinations: [M + H]^+^, [M + Na]^+^, [M + NH_4_]^+^, [2M + H]^+^, and [M + H − H_2_O]^+^. In ESI-mode, the following adducts were considered: [M − H]^−^ and [M + Cl]^−^. Both up- and down-regulated plasma lipids were identified in Progenesis QI by searching various databases, including HMDB, ChemSpider, and Lipid Maps, and reported on the basis of their fold-change from the control plasma and their statistical significance on the basis of ANOVA. Matching database entries were made on the basis of accurate mass, isotope similarity, where data were available, and fragmentation [65,66].

### 4.5. Gas Chromatography-Mass Spectrometry (GC-MS)

An Agilent 6890N gas chromatograph fitted with an Agilent 7683B liquid autosampler and an Agilent 5975B mass selective detector was used to analyse duplicate samples of the plasma (50 μL), which had been blown to dryness under N_2_ after the addition of ultrapure pyridine (100 μL; Merck, Darmstadt, Germany). Dry residues were derivatized with MOX followed by BSTFA/TMCS and analysed by GC-MS as described [28,67,68]. Data were collected from 12 to 55 min. Metabolites were identified by the comparison of their mass spectra with the NIST 14 spectral library comprising 242,466 GC-MS spectra. In addition, the chemical identity of a metabolite was confirmed by comparing its retention time (min) and mass spectrum with an in-house collection of 110 authentic metabolites.

### 4.6. Multivariate Data Analysis and Univariate Statistics

Deconvoluted data in Progenesis QI were imported into SIMCA 17 and subjected to an unsupervised Principal components analysis (PCA) followed by supervised partial least squares-discriminant analysis (PLS-DA) and orthogonal PLS-DA (OPLS-DA) [67]. Univariate statistical analysis was performed using GraphPad Prism 9.4.1 (Graph Pad Software, Inc., La Jolla, CA, USA). Means are expressed ± s.e.m., and *p*-values are all two-sided. Group differences were analyzed nonparametrically using Kruskal–Wallis for three or more datasets or the Mann–Whitney U-test for two datasets. In order to minimize false discovery, *p*-values derived from multiple comparisons were subjected to Dunn’s correction. These conservative measures [66] were adopted to minimize the likelihood that lipid biomarkers for HCV infection arose stochastically. Nonparametric Spearman rank correlation was used to correlate the fold induction for each gene studied by qRT-PCR with the viral load.

## Figures and Tables

**Table 1:** Plasma metabolites up- and down-regulated in HCV-positive patients determined by UPLC-ESI-QTOFMS.

**Metabolites Upregulated in HCV+ Patients**	***m*/*z***	**Adducts**	**Max Fold Up in HCV-Pos**
Azelaic acid	171.1033	M + H − H_2_O	2.7
Decanamide (10:0)	172.1686	M + H	2.8
Dodecanamide (12:0)	200.2004	M + H	2.3
Palmitamide (16:0)	255.2561n	M + H, M + Na	7.1
Palmitoleamide (16:1)	253.2404n	M + H, M + Na	14.3
Stearamide (18:0)	306.2750	M + Na	81.3
Oleamide (18:1)	GC-MS		8.0
Linoleamide (18:2)	279.2559n	M + H, M + Na	20.0
(5*Z*,8*Z*,11*Z*,14*Z*)-Eicosatetraenamide (20:4)	303.2537	M + H, M + Na	11.0
LTB4-ethanolamide (20:4)	402.2630	M + Na	4.6
Anandamide (20:4)	362.2118	M + Na	4.3
*N*-Linoleoyltaurine (18:2)	388.2509	M + H	2.0
Sphingosine 1-phosphate d18:1	362.2454	M + H − H_2_O	3.1
Sphingosine 1-phosphate d16:1	352.2252	M + H	2.0
Sphingosine 1-phosphate d19:1	416.2545	M + Na	5.3
Sphinganine 1-phosphate d18:0	382.2680	M + H	2.0
Pregnenolone sulfate	379.1956	M + H − H_2_O	2.6
Dolichol phosphate	463.2966	M + Na	3.0
PG(14:1(9*Z*)/12:0)	659.3857	M + Na	2.6
PS(*O*-16:0/12:0)	666.4694	M + H	3.7
PS(22:0/22:0)	904.7051	M + H	446
**Metabolites Downregulated in HCV+ Patients**	***m*/*z***	**Adducts**	**Max Fold Down in HCV-Pos**
TG(66:17)	1047.7374	M + Na	4.2
TG(42:1)	721.6286	M + H	18.0
PG(38:9)	771.4636	M + H − H_2_O	2.6
PC(16.1(9*Z*)/2:0)	535.3254n	M + H, M + H − H_2_O	3.1
PC(16:0/3:0)	550.3495	M − H	2.5
PS(13:0/18:4)	736.4173	M + Na	4.2
PS(*O*-16:0/20:0)	812.5563	M + Cl	12.3
PE(6:0/6:0)	412.2115	M + H	30.5
PE(18:4(6*Z*,9*Z*,12*Z*,15*Z*)/18:4(6*Z*,9*Z*,12*Z*,15*Z*)	754.4474	M + Na	2.0
LPC(3:1(2*E*))	312.1205	M + H	∞
LPC(16:0)	494.3204	M − H	2.4
LPC(*P*-19:1(12*Z*))	554.3234	M + Cl	2.1
LPC(20:2(11*Z*,14*Z*))	570.3500	M + Na	1.5
LPS(17:1(9*Z*))	492.2705	M + H − H_2_O	1011
LPS(22:2(13*Z*,16*Z*))	600.3262	M + Na	2.0
LPE(18:3(6*Z*,9*Z*,12*Z*))	458.2696	M + H − H_2_O	347
LPA(17:1(9*Z*))	445.2295	M + Na	4.7
LPA(17:2(9*Z*,12*Z*)	455.1969	M + Cl	3.5
LPA(20:3(8*Z*,11*Z*,14*Z*))	483.2455	M + Na	159
LPA(22:1(11*Z*))	515.3127	M + Na	2.0
LPA(22:4(7*Z*,10*Z*,13*Z*,16*Z*))	509.2618	M + Na	9.9
LPG(22:4(5*Z*,8*Z*,11*Z*,14*Z*))	555.2697	M + Na	48.6
LPI(20:2(11*Z*,14*Z*))	625.3370	M + H	∞
LPI(20:5(5*Z*,8*Z*,11*Z*,14*Z*,17*Z*))	619.2895	M + H	461
LPI(21:0)	643.3766	M + H	3.3
LPI(22:4(7*Z*,10*Z*,13*Z*,16*Z*))	631.3230	M + H − H_2_O	161
LPI(22:6(4*Z*,7*Z*,10*Z*,13*Z*,16*Z*,19*Z*))	645.3042	M + H	2879
*N*-Acetylsphingosine 1-phosphate	444.2489	M + Na	∞
Anandamide *O*-phosphate	410.2431	M + H − H_2_O	2.4
Glycochenodeoxycholic acid 7-sulfate	552.2596	M + Na	184
Cholic acid glucuronide	585.3295	M + H	5.4
Lipoxin C4	640.2959	M − H	2.5
NeuAcα2-3Galβ-Cer(d18:1/16:0)	1013.6566	M + Na	3.4
NeuAcα2-3Galβ-Cer(d18:1/20:0)	1069.7198	M + Na	2.8

n—neutral mass; GC-MS—determined by gas chromatography-mass spectrometry. Shading indicates negative ions (M − H; M + Cl). All other ions are positive ions (M + H; M + Na; M + H − H_2_O).

## Data Availability

The authors declare that all data supporting the findings of this study are available within this paper or can be obtained from the corresponding authors upon request.
